# Physiotherapy practice in lymphoedema in South Africa: A survey

**DOI:** 10.4102/sajp.v79i1.1907

**Published:** 2023-10-27

**Authors:** Carys A. Rhodes, Corlia Brandt, Monique Keller

**Affiliations:** 1 Department of Physiotherapy, Faculty of Health Sciences, University of the Witwatersrand, Johannesburg, South Africa

**Keywords:** lymphoedema, physiotherapy, treatment, guidelines, South Africa

## Abstract

**Background:**

Lymphoedema is a chronic condition that is increasing in prevalence and requires specialised management to avoid possible life-threatening complications.

**Objectives:**

To describe the perceived knowledge of physiotherapists about lymphoedema and its management, the lymphoedema patient load seen by physiotherapists, and the current treatment approaches of physiotherapists in South Africa when managing lymphoedema.

**Method:**

A quantitative study using self-administered, online questionnaires were distributed among physiotherapists.

**Results:**

Knowledge of lymphoedema management is perceived to be lacking among physiotherapists. Physiotherapists are getting limited referrals and spending little time managing patients with lymphoedema. Only a few physiotherapists have post-graduate education in lymphoedema management, thus international treatment standards still need to be met.

**Conclusion:**

In order to meet international standards and patient needs, future research investigating the physiotherapy perspective is needed in lymphoedema management.

**Clinical implications:**

Educational bodies and policymakers may use this data to facilitate improvement in physiotherapy management of the condition and provision of care.

## Introduction

Lymphoedema is a chronic condition characterised by the progressive swelling of one or more limbs, trunk, head, neck or genitalia, and can be from a primary (congenital) or secondary cause. The condition’s prevalence is estimated to be 140 to 200 million people worldwide (Gerez, Horibe & Ferreira 2020), but as an under-reported and under-diagnosed condition, this number could be much higher (Rockson & Rivera 2008). The exact number of people with lymphoedema in South Africa is unknown, but it is estimated that around 1.3 m people have the condition in South Africa, based on the World Health Organization (WHO) statistics released in 2014 (Herbst 2021).

Treatment of lymphoedema includes both conservative and surgical interventions. Conservative treatments such as complete decongestive lymphatic therapy (CDLT), which includes manual lymph drainage, compression therapy by bandaging and garment prescription, remain the most common form of treatment for lymphoedema (Fialka-Moser et al. 2013). Conservative management in the form of CDLT, by a healthcare professional trained in lymphoedema management (at an undergraduate or postgraduate level) remains the gold standard (Bahtiyarca et al. 2019). Lymphoedema management falls under the scope of physiotherapy in South Africa and thus implies that it should be included in undergraduate physiotherapy curricula.

Lymphoedema has far-reaching implications not only for the patients with the condition but also for global economies. If left untreated, lymphoedema can result in cellulitis, lymphangitis and in rare cases lymphangiosarcoma (Agbenorku 2014). These conditions can lead to severe illness, amputation, and even death. Patients with lymphoedema can also suffer from psychological problems and a decreased quality of life related to living with the condition (Tidhar & Armer 2018; Wedin et al. 2019). The economic burden on patients with lymphoedema cannot be overlooked, as the condition affects productivity, leads to time off work, and can be expensive to manage. This places a financial strain on patients with the disease and the healthcare system (Eneanya, Garske & Donnelly 2019). In a developing country such as South Africa, where the economy and healthcare system are already strained, an increase in a population struggling with unmanaged lymphoedema could have dire consequences.

Cancer and the treatment thereof remain one of the leading causes of secondary lymphoedema. The disease and treatments such as surgery and radiation, can result in scarring, fibrosis or complete destruction of lymphatic tissue, leading to lymphoedema (Koca, Aktaş & Kurtgil 2020). With the advancement of modern medicine and specifically the treatment of cancer becoming more effective, the number of people affected by this condition will continue to grow (Wolfs et al. 2020). The number of new cancer cases in South Africa in 2020 was 108 168 (‘IARC Globocan, South Africa Fact Sheet’, 2020) and this number could indicate a potentially growing population with cancer-related lymphoedema in South Africa. Khutjwe (2018) found that 29.7% of the 155 female patients undergoing radiotherapy for gynaecological cancer in a Johannesburg hospital developed lower limb lymphoedema.

Research into the lymphoedema field remains limited, compared with other major illnesses and diseases. Most literature on lymphoedema is patient-centred, focusing on the efficacy of treatment, different treatment modalities, prevention of the condition (Stuiver et al. 2015), quality of life, and patient expectations (Tidhar & Armer 2018). Continued research into managing lymphoedema from a practitioner’s point of view can yield invaluable data on current practice standards, prevalence, shortcomings, advancements, and needs within the field. Studies conducted internationally by Anderson et al. (2019), Armer et al. (2010), Fialka-Moser et al. (2013), Moffatt, Doherty and Morgan (2003), and Schulze et al. (2018) are examples of quantitative studies that focused on health professionals’ management of patients with lymphoedema. These studies described the treatment approaches of lymphoedema therapists and physicians, and provided data on epidemiology within their patient population. Our study aims to provide an overview of contemporary physiotherapy practice in managing lymphoedema in a South African context by investigating perceived knowledge, patient load, and current treatment approaches of physiotherapists.

## Methods

Our study was cross-sectional in design and quantitative data were collected using an online, self-administered questionnaire. The questionnaire was completed online by physiotherapists in South Africa. Physiotherapists registered with the Health Professions Council of South Africa (HPCSA) provided a population of 8 058 participants (HPCSA 2020). Non-probability convenience sampling was used. The sample size for our study was calculated according to guidelines set out by Cochrane (Sugden, Smith & Jones 2000) with a confidence level of 95% and an alpha level of 5%. The required sample size for our study was 367 participants. This calculation was performed on Raosoft online sample size calculator (Raosoft 2014). To participate in the online questionnaire, participants had to be physiotherapists with a university degree in physiotherapy, working at government or private institutions, hospitals or clinics when the questionnaire was completed and understand and respond to questions posed in English. The participants were excluded if they were not practising physiotherapy in South Africa.

### Data collection tool

The first author developed the questionnaire using Research Electronic Data Capture (REDCap) software, version 11.2.2 (Harris et al. 2009, 2019). It consisted of open- and closed-ended type questions in five sections. The questionnaire covered the following topics to meet the research objectives: the participants’ perceived knowledge of lymphoedema management, details on patient load, such as the number and type of patients managed and lymphoedema management approaches. Section one and two, on demographics and perceived knowledge, was completed by all respondents. Sections three, four and five were only completed by respondents who identified themselves as managing patients with lymphoedema.

The development of the questionnaire and the use of Likert scale-type questions were informed by reviewing other questionnaires from studies with similar study designs (Gracey, McDonough & Baxter 2002; Grieve & Palmer 2017; Reeve, Denehy & Stiller 2007; Shimpi et al. 2014). Studies with similar aims and objectives such as the American Lymphoedema Framework Project online survey (Anderson et al. 2019) and the surveys used in *‘Lymphoedema therapists: A national and international survey’* by Davies et al. (2012) were also considered when drafting the questions. The questionnaire content was guided by the ‘Best practice for the management of Lymphoedema: International consensus’ compiled by the Lymphoedema Framework (Moffatt et al. 2006) to ensure that the questions reflected current clinical guidelines. Five ‘experts’ in the field who had completed the 135 h certification in lymphatic therapy and are active members of the Lymphoedema Association of South Africa (LAOSA) were consulted during the development of the questionnaire and piloted the questionnaire to assist in determining content validity. (Lymphoedema Association of South Africa, n.d.)

### Procedure

Various databases were used to distribute the questionnaire to physiotherapists matching the inclusion criteria. The South African Society of Physiotherapy (SASP) emailed all its members with an advertisement to request participation in our study and the link to the questionnaire. Emails with the advertisement were also sent to members of the LAOSA, whose details are freely available on the LAOSA website. Platforms such as Facebook and WhatsApp were used to share the advertisement and link to various groups that have physiotherapists as their members. The advertisement invited physiotherapists to participate and included a brief overview of our study and inclusion criteria. The link provided in the advertisement directed participants to the questionnaire on the REDCap site. Included in the link to the questionnaire was a study information document explaining the purpose of our study and their rights regarding participation. Reminders were posted on the social media platforms every 2 weeks. The link was made available for 3 months after the onset of our study.

### Data analysis

Data from the questionnaire were collected on REDCap and exported to Microsoft Excel, version 2111, in raw data format. The first author checked the data and coding and then exported data to Statistical Package for the Social Sciences (SPSS), version 27 (IBM Corp. Released 2020. IBM SPSS Statistics for Windows, version 27.0. Armonk, New York), for analysis. Descriptive, non-parametric analysis was performed on the nominal and ordinal data. Mean scores, frequency distributions, percentages and ratios were used to analyse and describe the continuous data.

### Ethical considerations

Ethical clearance for our study was obtained from the Human Research Ethics Committee (Medical) of the University of the Witwatersrand before the start of our study (ethical clearance number M200540). The questionnaire took approximately 10–20 min to complete. There was neither any risk to the participants in our study nor any direct benefit to the participants before commencement of our study. Consent to participate in the online questionnaire was agreed to by all the participants before they could continue with the questionnaire. The identity of the participants remained confidential. Each participant was assigned a unique identifying number when completing the survey.

## Results

### Participants

A total of 402 qualified physiotherapists who currently work in South Africa completed the online self-administered questionnaire, which met the required sample size. Table 1 shows an overview of participants’ characteristics, shown by the most selected choices for each descriptor. A total of 153 respondents (43.5%) confirmed that they manage patients with lymphoedema in their day-to-day practice. In all 146 responses were captured regarding the level of education received in lymphoedema management. Most of the training received by these physiotherapists was clinical training on the job (43%, *n* = 63) or tuition received at an undergraduate level (26%, *n* = 38). Only 23 participants (15.8%) had completed thefurther certified 135-h training in lymphoedema management at a post-graduate level.

**TABLE 1:** An overview of participants’ characteristics, shown by the most selected choices for each descriptor.

Characteristic category	Total responses	Number and percentage of most chosen descriptor		Descriptor
		*n*	%	
Current clinical setting	402	284	70.6	Private outpatient practice
Years of experience in physiotherapy	389	136	35.0	More than 15 years of experience
Province in which they work	389	168	43.3	Gauteng
Size of the community serviced	388	234	60.3	Large urban area > 125 000 people

### Perceived knowledge of physiotherapists on lymphoedema management

**FIGURE 1:**
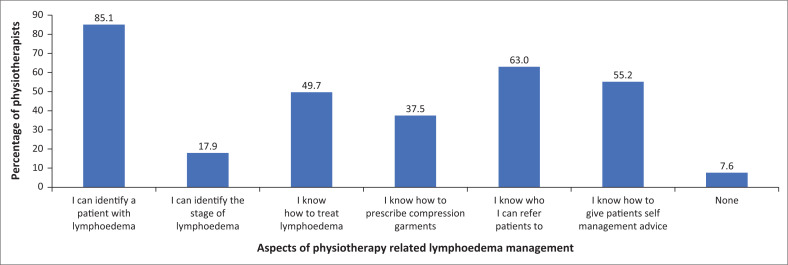
Physiotherapist’s self-reported knowledge of the different aspects of physiotherapy-related lymphoedema management (*n* = 367).

The perceived knowledge of physiotherapists regarding the pathology, characteristics, causes, stages, risk factors, treatment options, and complications relating to lymphoedema was rated on a scale from no knowledge to expert, as per the Likert scale rating discussed in the methodology section. Forty respondents did not complete this section entirely. However, responses showed that physiotherapists consistently rated their knowledge as ‘fair’, with 46.1% (*n* = 167) of the 362 physiotherapists who answered this section indicating that their knowledge of the stages of lymphoedema was poor. Physiotherapists were asked to report on their perceived knowledge of different aspects of lymphoedema management, including patient identification, staging of the condition, ability to treat the condition, prescription of compression garments and ability to give self-management advice, as shown in Figure 1. Most (85.1%, *n* = 313) of the physiotherapists reported that they could identify a patient with lymphoedema but only 49.7% (*n* = 183) said that they knew how to treat the condition. Out of 367 participants who answered this section, 357 (97.3%) indicated they knew that physiotherapists could offer lymphoedema management.

### The lymphoedema patient load being seen by physiotherapists in a clinical setting

#### Number of patients

In all, 148 physiotherapists who identified themselves as managing patients with lymphoedema completed the question on sources of referrals. A total of 61 (51.4%) patients who come to physiotherapy for management do so by self-referral. Physiotherapists also get referrals from oncologists (44.6%, *n* = 66), general practitioners (41.9%, *n* = 62), surgeons (38.5%, *n* = 57), fellow physiotherapists (24.3%, *n* = 36), other healthcare professionals (19.6%, *n* = 29), and vascular surgeons (18.2%, *n* = 27). Out of 146 respondents, 89 (61.0%) reported receiving fewer than one new lymphoedema referral in a month, with 32.2% (*n* = 47) receiving one to five new referrals in a month.

**FIGURE 2:**
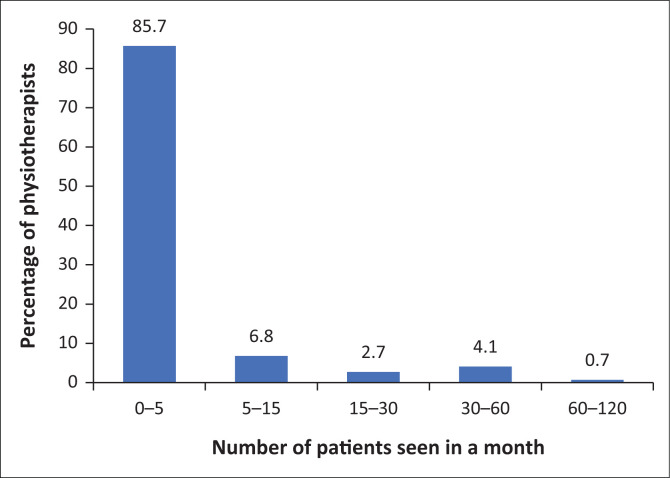
The number of patients with lymphoedema seen in a month by physiotherapists (*n* = 146).

Of 150 responses, 16.7% (*n* = 25) identified themselves as working in a practice with a special interest in treating patients with lymphoedema. While evaluating how much of their clinical time is taken up with lymphoedema management, 85% (*n* = 125) of 147 respondents reported that less than 20% of their average workday was spent managing patients with lymphoedema. Figure 2 shows that regarding the number of patients seen, 85.7% (*n* = 126) of physiotherapists are seeing 0–5 lymphoedema patients a month.

#### Types of patients managed

Paediatric patients (under 12 years old) are managed by 7.4% (*n* = 8) of 107 respondents, and adolescents (12–18 years old) are managed by 19.6% (*n* = 21) of 108 respondents. The dominant patient population groups managed by the respondents are adults (18–65 years old) and geriatric patients (older than 65 years). Half (55.9%) of the 143 respondents (*n* = 80), reported that managing adult lymphoedema patients makes up more than half of their current patient load. In all, 47.6% (*n* = 62) of 130 respondents said that managing geriatric lymphoedema patients makes up more than half of their current patient load.

**FIGURE 3:**
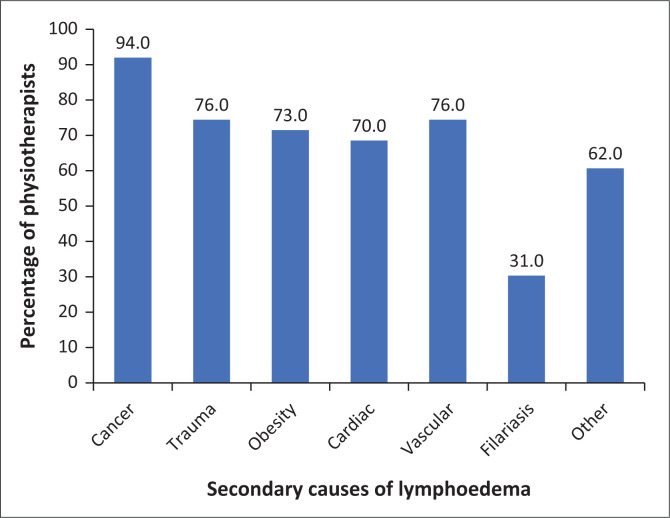
The causes of secondary lymphoedema as seen by physiotherapists.

Primary lymphoedema is managed by 59% (*n* = 49) of 83 respondents, with 36% (*n* = 30) reporting that these patients comprise less than a quarter of their current patient load. Cancer and vascular disease are the most common causes of secondary lymphoedema seen by physiotherapists as shown in Figure 3.

For physiotherapists managing patients with lymphoedema, the most commonly treated area of lymphoedema out of 137 responses was identified as the upper limb (94.2%, *n* = 129) and the lower limb (95.3%, *n* = 122). Table 2 shows the other anatomical areas that physiotherapists are treating for lymphoedema.

**TABLE 2:** The distribution of anatomical areas treated for lymphoedema, represented as a percentage of the total patient load seen by physiotherapists.

Anatomical area	Total (*n*)	None (%)	1–25 (%)	25–50 (%)	50–75 (%)	75–100 (%)
Upper limb	*137*	5.8	22.6	20.4	27.7	23.4
Lower limb	*128*	4.7	25.8	25.8	28.9	14.8
Head and neck	*80*	58.8	28.8	5.0	3.8	3.8
Trunk	*79*	58.2	26.6	10.1	1.3	3.8
Genitalia	*69*	65.2	27.5	5.8	1.4	0.0

### Current physiotherapy practice in the management of lymphoedema

**FIGURE 4:**
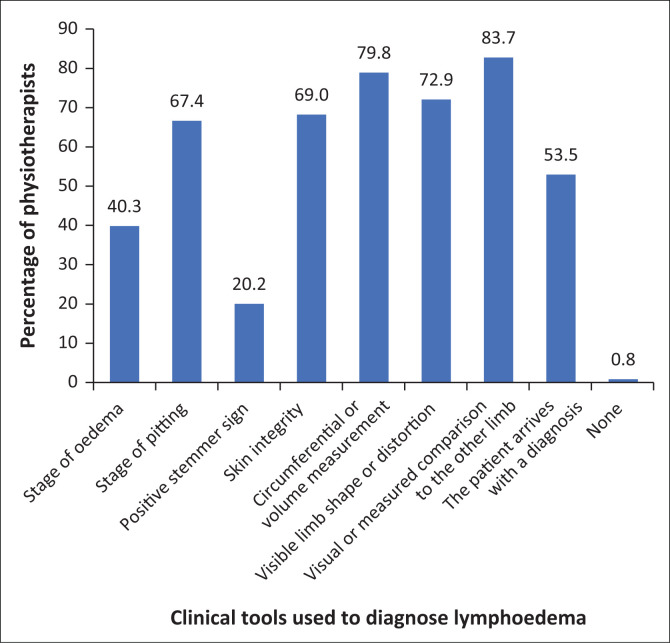
The clinical tools physiotherapists identified for use in the diagnosis of lymphoedema (*n* = 129).

A total of 129 physiotherapists who identified themselves as managing patients with lymphoedema answered a question on what diagnostic tools they use daily for patients with lymphoedema. Figure 4 shows the results. Comparison to the other limb, visual or measured is most used (83.7%, *n* = 107) followed by circumferential or volume measurements (79.8%, *n* = 102). In 53.5% (*n* = 68) of cases, the patient already has a diagnosis when presenting for physiotherapy management.

**FIGURE 5:**
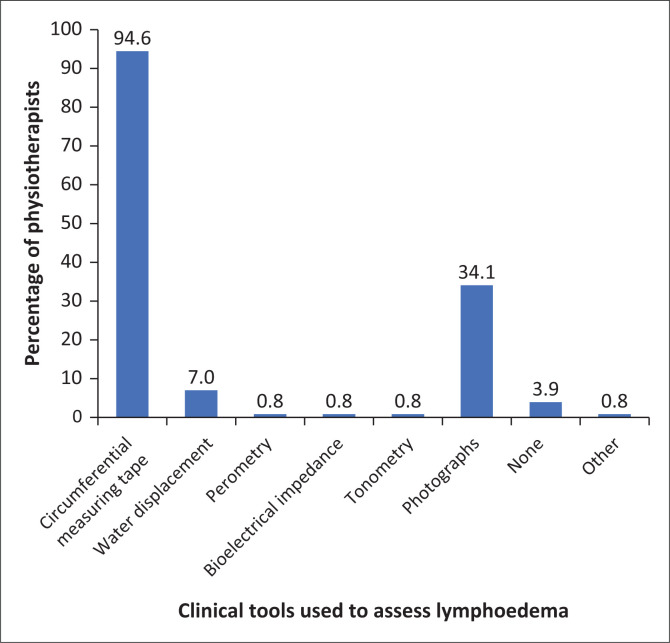
The clinical tools physiotherapists identified using in the assessment of lymphoedema (*n* = 129).

Figure 5 shows the clinical tools that physiotherapists use to assess lymphoedema. Circumferential tape measurements (94.5%, *n* = 122) photographs for comparison (34.1%, *n* = 44) and water displacement (6.9%, *n* = 9) are the most common lymphoedema assessment methods utilised by the 129 respondents.

**FIGURE 6:**
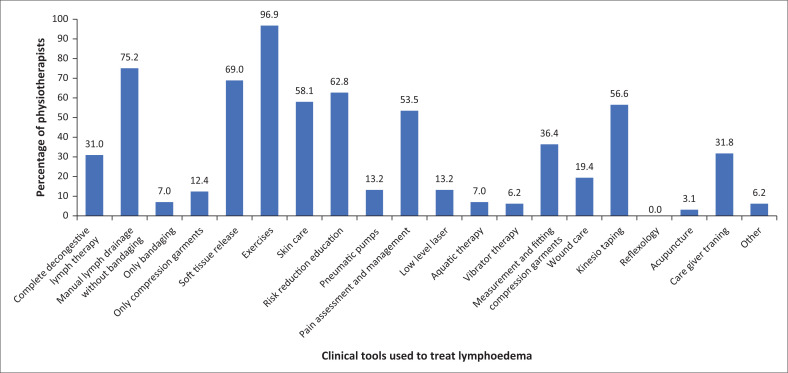
The clinical tools physiotherapists identified for use in the treatment of lymphoedema (*n* = 129).

Figure 6 shows the clinical tools that physiotherapists reported using when treating lymphoedema. Only 31% (*n* = 40) of the 129 physiotherapists offered CDLT. Other treatment techniques used included exercises, soft tissue release and Kinesio Taping method.

## Discussion

### Perceived knowledge of physiotherapists on lymphoedema management

Physiotherapists in South Africa seem to have a fair understanding of the management of lymphoedema, based on the results from the questionnaire. While a small percentage of the respondents rate their perceived knowledge as ‘good’ or even ‘expert’, the majority of respondents showed a worrying lack of perceived knowledge on important topics such as pathology, characteristics, causes, stages, risk factors, treatment options, and complications relating to lymphoedema. Although 97.3% of physiotherapists in our study (*n* = 357) indicated that they knew that physiotherapists could offer lymphoedema management, only 49.7% (*n* = 183) knew how to treat the condition. This echoes findings by international studies, such as Davies et al. (2012) and Gerez et al. (2020) who found that when investigated, the perceived knowledge of healthcare professionals about lymphoedema and aspects of lymphoedema, management was lacking.

The dearth of knowledge among physiotherapists about lymphoedema could be explained by the level of education received on the topic, both at an undergraduate level and at a postgraduate level or it could be because of the disinterest of physiotherapists in treating the population and furthering their education in the field. The minimum training standards for physiotherapists in South Africa, as set out by the HPCSA, require undergraduate training in oedema assessment and management. However, comprehensive management of lymphoedema remains a post-graduate course (‘HPCSA. Professional Board For Physiotherapy, Podiatry and Biokinetics. Minimum Standards of Training: Physiotherapy’, 2023).

The lack of comprehensive education and awareness about lymphoedema at an undergraduate level can foster a worrying cycle of ignorance and stagnation in the field of lymphoedema management in South Africa. Schulze et al. (2018) suggest that a lack of comprehensive education on the topic at a university level can lead to therapists needing to be made aware of the benefit of treating the condition and, therefore lead to them not pursuing it in their career. If more physiotherapists fulfil the role of lymphoedema management, their role within the multidisciplinary team will continue to exist.

### Lymphoedema patient load being seen by physiotherapists in a clinical setting

Physiotherapists are, however, still receiving referrals of patients for lymphoedema management from multiple multidisciplinary team members. Oncologists, general practitioners, surgeons and other healthcare professionals refer patients. This indicates that a multidisciplinary approach to lymphoedema management is needed, where physiotherapists are regarded as key role players. However, 61% of physiotherapists report receiving fewer than one new lymphoedema referral in a month (*n* = 146). Although patients with lymphoedema are being referred to physiotherapy for treatment, the number of patients who are being referred is surprisingly low considering the high estimated prevalence of this condition in South Africa. It appears that a poor understanding of lymphoedema as a condition and possible management options within the healthcare profession, as well as a lack of knowledge and awareness in the patient population are contributing to this discrepancy.

There could be a large percentage of patients with lymphoedema who need to receive the correct treatment. This reflects findings from a study by Keast et al. (2015), which found that providing care to all patients with possible lymphoedema is not being provided. This leads to a perception that a lack of awareness within the healthcare profession may be the culprit for poor referral rates. Rockson and Rivera (2008) reported similar findings and discussed that healthcare professionals continue to misdiagnose patients with lymphoedema, resulting in the patients receiving incorrect care.

With poor referrals, lymphoedema patients make up very little of physiotherapists’ patient load in a month across South Africa. Most physiotherapists (83.3%) work in a more generalised practice (*n* = 150), and the time dedicated to managing patients with lymphoedema takes up less than 20% of their average workday. This could be because the patient load is being distributed among other healthcare professionals such as nurses, occupational therapists and massage therapists who also have a role in lymphoedema management, as was noticed by Anderson et al. (2019). With so few lymphoedema patients being seen by physiotherapists, it seems reasonable that physiotherapists are not spending much time developing their interest and skill set in the field.

Physiotherapists predominantly manage patients with lymphoedema who are older than 18 years of age. Only a few physiotherapists are seeing children and adolescents for lymphoedema management. This is in keeping with the fact that paediatric lymphoedema is quite rare and poorly identified, as reported by Connell et al. (2014) and Todd et al. (2014).

Of the physiotherapists managing lymphoedema patients, most of the patient load is made up of lymphoedema from a secondary cause. This is seen in other studies by Anderson et al. (2019) and Armer et al. (2010) who also found that more than 80% of the patients seen by lymph therapists were of a secondary cause. While 59% (*n* = 83) of physiotherapists identify managing primary lymphoedema patients, they make up a small percentage of their patient load. Consistently across the literature, cancer is the biggest cause of secondary lymphoedema in patients seen by therapists. The same is found within this data set, with 93.8% of 131 participants identifying cancer as the biggest cause of their patients’ lymphoedema. This is unsurprising as worldwide improvements in oncological medical care result in an increase in cancer survivors and secondary complications such as lymphoedema (Siegel, Miller & Jemal 2020).

The data show that physiotherapists see a broad range of patients with lymphoedema of the upper limb, lower limb, trunk, head and neck, and genitalia. However, lymphoedema of the upper and lower limb are the most common areas treated, as seen in other practitioner surveys by Anderson et al. (2019) and Armer et al. (2010). Anderson et al. (2019) reported that 53% of patients treated by the therapists in their study (*n* = 950) had upper limb lymphoedema and 30% had lower limb lymphoedema. Similarly, Armer et al. (2010) found that 59% of patients treated by respondents in their study (*n* = 415) had lymphoedema of the upper limb and 30% of the lower limb. Upper limb lymphoedema greatly impacts functional ability in terms of activities of daily living and in the workplace. Patients with upper limb oedema often struggle with psychological and social issues from living with the condition (Koca et al. 2020).

### Current physiotherapy practice in the management of lymphoedema

While looking at the current practices of physiotherapists in the management of lymphoedema, the results of our study were compared with international standards set by different guidelines such as the International Lymphoedema Framework (Moffatt et al. 2006) and the ONS Guidelines (Armer et al. 2020).

Methods of diagnosis and assessment of the oedematous area tend to be prone to subjectivity and are easy to perform. Visual or measured comparison to the opposite limb, observation of skin integrity, and circumferential or volume measurements are all routinely used. Although these methods may be unreliable, they are recognised as being the most commonly used in the international literature and guidelines because of their ease of use (Moffatt et al. 2006). Other objective measures such as lymphoscintigraphy and indocyanine green (ICG) fluorescent lymphography are not readily available in many settings and expensive to use (Torgbenu et al. 2023).

With regard to the current treatment techniques used by physiotherapists in South Africa, we see that very few therapists follow international guidelines. Only 40 of 129 (31%) physiotherapists use CDLT to manage patients with lymphoedema. This is a concerning finding with CDLT being the gold standard of lymphoedema management as supported by Armer et al. (2020) and Marco, Pillay and Schoonheim (2014). It seems that physiotherapists need to have the correct education or skill set to manage lymphoedema appropriately. This finding follows with the fact that only a few physiotherapists move into lymphoedema management as a special interest in South Africa and continue their post-graduation into the field (15.8%, *n* = 146). Most physiotherapists managing lymphoedema are providing treatment based on their knowledge received in the ‘on the job’ training and undergraduate training. Treatment such as exercise prescription (96%, *n* = 129), MLD without bandaging (75.1%, *n* = 129), soft tissue release (68.9%, *n* = 129), risk reduction education (62.7%, *n* = 129), skin care (58.1%, *n* = 129), KT (56.5%, *n* = 129), and pain management techniques (53.4%, *n* = 129) are being used. Although all of the aforementioned modalities offer value to the patient, and a few of them make up the components of CDLT, the results show that there needs to be an improvement in the overall education of physiotherapists about lymphoedema management to standardise care to patients, improved treatment efficacy and meet international guidelines.

### Strengths and limitations

Our study design allowed a wide variety of physiotherapists to have access to participation in our study, which improved the generalisability of the findings to the South African physiotherapy community. This strengthened our study. A limitation of our study was the inconsistent response rate on some of the questions in the questionnaire.

### Implications and considerations

The lack of highly qualified lymphoedema physiotherapists with a special interest in managing lymphoedema points to a few fundamental problems that need to be addressed. There needs to be an emphasis on educating all physiotherapists to bridge the gap between physiotherapists with a special interest in lymphoedema who are experts in the field and the generalists who know very little. Educational institutions should be approached with clear evidence of the need for education in this field to develop a way forward, starting with the fundamentals of lymphoedema at an undergraduate level. When this becomes a reality, and more physiotherapists are moving into the field, awareness of the condition will grow among the medical community and the patient population with lymphoedema. We can then strive to achieve international standards of care.

## Conclusion

The lack of perceived knowledge in the field, the poor referral rate, and the inadequate management of patients with lymphoedema in South Africa highlight that more needs to be carried out to promote the management of lymphoedema. There appears to be a diverse range of patients who require care, but at this stage, very few physiotherapists are available to offer the gold standard of lymphoedema management. This study shows that more work needs to be performed in South Africa in the field of lymphoedema management in terms of education, awareness and access to care from a physiotherapy perspective.
